# Rehabilitation in Iran's Health System: A Qualitative Inquiry Into Policy Landscape and System Strengthening Pathways

**DOI:** 10.1002/hsr2.72001

**Published:** 2026-05-11

**Authors:** Leila Doshmangir, Bahram Amirshakeri, Maryam Ghiassipour, Roghayeh Khabiri, Reza Majdzadeh

**Affiliations:** ^1^ Social Determinants of Health Research Centre Tabriz University of Medical Sciences Tabriz Iran; ^2^ Department of Health Policy & Management, School of Management & Medical Informatics Tabriz University of Medical Sciences Tabriz Iran; ^3^ Department of Physiotherapy, School of Rehabilitation Sciences Tabriz University of Medical Sciences Tabriz Iran; ^4^ Supreme Council for Health and Food Security Ministry of Health & Medical Education Tehran Iran; ^5^ Tabriz Health Services Management Research Centre Tabriz University of Medical Sciences Tabriz Iran; ^6^ School of Health and Social Care University of Essex Colchester UK

**Keywords:** health policy, health services, health system, Iran, policy interventions, rehabilitation, universal health coverage

## Abstract

**Background and Aims:**

Rehabilitation is essential in supporting individuals with disabilities to improve their functional abilities and independence, and it is considered a key element of universal health coverage (UHC). This study examines the current considerations within Iran's rehabilitation system and offers policy suggestions aimed at strengthening and improving its performance.

**Methods:**

This qualitative study utilized a systematic review of relevant documents, 22 in‐depth interviews, and two focus group discussions (FGDs) with 24 participants from different levels of the health system. Participants included policymakers from the Ministry of Health and the Social Security Organization, rehabilitation center managers, and academic faculty. Data were thematically analyzed using MAXQDA version 22.

**Results:**

Rehabilitation services in Iran have not yet been fully incorporated into health policy and planning, which has resulted in opportunities for further development in areas such as accessibility, service availability, governance structures, stakeholder coordination, and workforce training. The current governance arrangements and intersectoral collaboration mechanisms may benefit from greater coherence to enhance overall system performance. Moving toward a more unified stewardship framework under the Ministry of Health could contribute to aligning policies and strengthening cross‐sectoral coordination. Furthermore, integrating rehabilitation services into primary healthcare represents an important step toward providing comprehensive and continuous care. Designing an evidence‐based integration model will require a systematic assessment of existing capacities and resources.

**Conclusion:**

Enhancing Iran's rehabilitation system can benefit from context‐sensitive and evidence‐informed policy initiatives. Recognizing rehabilitation as an integral component of universal health coverage may support more equitable access, continuity of services, and improved health outcomes for individuals who require such care.

## 1 Introduction

Expanding rehabilitation services is essential for achieving universal health coverage (UHC), as it enhances quality of life, improves health outcomes, and increases the cost‐effectiveness of healthcare services [1]. Rehabilitation is a fundamental component of modern health systems, addressing the diverse needs of individuals across all stages of life. The United Nations highlights the importance of equitable access to high‐quality rehabilitation services while protecting individuals from catastrophic health expenditures [2].

Globally, the demand for rehabilitation services has grown due to demographic changes, including population aging and the rising prevalence of chronic diseases [3]. Additional factors such as conflicts, natural disasters, congenital disorders, movement impairments, mental health conditions, and occupational injuries further contribute to the increasing need for rehabilitation [4]. Sedentary lifestyles and prolonged use of digital devices also exacerbate health issues such as musculoskeletal disorders, sleep disturbances, and obesity. Meanwhile, technological advancements including robotics, artificial intelligence, and virtual reality along with greater societal awareness, have driven higher utilization of rehabilitation services.

Rehabilitation aims to enhance functional independence and quality of life, regardless of the underlying condition or disability. It spans a continuum of care, from prevention to therapeutic and restorative interventions [5]. Effective rehabilitation requires integration at all levels of the health system, ensuring early identification of needs and continuity of care throughout the recovery process [6]. However, many countries, particularly in the Middle East, struggle with insufficient resources for disability diagnosis and prevention, inadequate rehabilitation infrastructure, and limited access to specialized services [7, 8]. Additionally, fragmented governance, inefficient organizational structures, and insufficient funding hinder rehabilitation service delivery [9, 10]. In response to these challenges, the World Health Organization (WHO) launched the Rehabilitation 2030 initiative, drawing international attention to the substantial unmet need for rehabilitation services. This initiative advocates for strengthening health systems to provide equitable rehabilitation care, particularly in low‐ and middle‐income countries. Key objectives include expanding service access, integrating rehabilitation within broader health and social services, enhancing economic and social support for individuals with disabilities, and strengthening the rehabilitation workforce through education and training [11]. The initiative also emphasizes advancing research and innovation to ensure rehabilitation becomes an integral part of UHC and improves the quality of life for people with disabilities [12].

During recent decades, Iran has experienced ongoing opportunities for further development in the delivery of rehabilitation services [7, 8]. Since the expansion of its public health network in 1985 and hospital management reforms in 1995, Iran's health policies have primarily focused on prevention and treatment [13]. In recent years, factors such as the COVID‐19 pandemic, fluctuations in access to certain medical supplies, changing trends in disease patterns including rising rates of cancer and chronic non‐communicable diseases along with constraints in healthcare expenditure and pressures on health insurance funds, have influenced the pace of development and scaling of rehabilitation services [14, 15].

Rehabilitation services in Iran are delivered across multiple levels by various institutions, though the degree of systematic coordination among them varies. This has contributed to a structure in which service organization and delivery can appear somewhat dispersed across stages ranging from policymaking to implementation. Inpatient rehabilitation services are offered in a number of specialized hospitals, while outpatient services including physiotherapy, occupational therapy, speech therapy, audiology, and orthotics/prosthetics are provided through both the Ministry of Health and the Welfare Organization. Additionally, community‐based rehabilitation initiatives, such as home care and vocational training, are also available. Strengthening linkages among these service components could support greater continuity of care and improve overall service efficiency [1, 16].

Current demographic and epidemiological patterns in Iran indicate a growing need for rehabilitation services. Factors such as an aging population, the increasing incidence of chronic conditions including cardiovascular and neurological diseases as well as road traffic injuries, mental health concerns, and developmental disorders contribute to this demand [17]. Existing legislation provides war veterans with access to rehabilitation services, and optimizing the availability of assistive technologies could further enhance the quality and continuity of care for this group, highlighting an area for ongoing system development [18].

Over the past decade, the relative share of rehabilitation in total health expenditure has experienced a decline, reflecting opportunities for more balanced resource allocation [19]. Currently, public rehabilitation facilities are limited in meeting the growing demand, and service availability tends to be concentrated in certain areas, which may affect equitable access [20]. Health insurance coverage for rehabilitation services in public facilities is partial, resulting in additional out‐of‐pocket expenses for many individuals. Rehabilitation services in the private sector are generally more costly, which can limit accessibility for some populations [19, 21].

Despite ongoing areas for development, Iran has increasingly recognized the role of rehabilitation in supporting universal health coverage and has introduced policy initiatives to enhance access and promote equitable service delivery. Rehabilitation continues to represent a component of the health system with potential for further growth. Strengthening this sector will benefit from a thorough understanding of current system dynamics and the design of context‐sensitive policy interventions to support the effective development of rehabilitation services.

## 2 Methods

### 2.1 Study Design

This qualitative study employed 22 in‐depth interviews and two focus group discussions (FGDs) with a total of 24 participants to comprehensively explore diverse perspectives on rehabilitation context and possible interventions for strengthening the system. The combination of in‐depth interviews and FGDs was chosen to capture both individual experiences and detailed insights (through interviews) as well as group dynamics and shared perspectives (through FGDs), thereby enhancing the depth and breadth of the findings.

Participants were purposively selected to ensure diversity in professional background, expertise, and organizational roles, as they were directly involved in or knowledgeable about rehabilitation services and policies. The sample size was determined based on the principle of data saturation, where no new themes emerged in later interviews.

The participants' ages ranged from 40 to 76 years, representing mid‐career to senior professionals. Interviews were conducted by the first and second authors using a semi‐structured interview guide developed based on the research objectives and key themes identified through the literature review. All interviews and FGDs were audio‐recorded with consent, transcribed verbatim, and analyzed using an inductive thematic analysis approach to derive codes and categories, which were subsequently organized into themes and sub‐themes related to rehabilitation challenges.

### 2.2 Setting and Participants

Participants were selected at the national level using a purposeful sampling method with maximum variation, considering their first‐hand experience, perspectives, and willingness to participate. Data collection continued until data saturation was reached. The study includedknowledgeable individuals, including policymakers from the Ministry of Health& Medical Education (MoHME) and Social Security organizations, State Welfare Organization, academic staff, rehabilitation service providers, and health experts with extensive experience and expertise in Iran's rehabilitation system. Tables 1 and 2 presents the characteristics of the participants, including their age range, organizational affiliations and fields of activity.

**Table 1:** Characteristics of interview participants (*n* = 22).

Participant No.	Age range (years)	Field of activity/expertise	Organization/Institution
1	35–45	Social Medicine	State Welfare Organization
2	40–55	Rehabilitation Management	State Welfare Organization
3	30–45	Occupational Therapy	State Welfare Organization
4	45–60	Rehabilitation Policy	State Welfare Organization
5	35–50	Physiotherapy Services	State Welfare Organization
6	35–50	Health Policy Research	University of Medical Sciences
7	30–55	Rehabilitation Research	University of Medical Sciences
8	40–55	Public Health Policy	University of Medical Sciences
9	35–50	Health Governance Research	University of Medical Sciences
10	45–60	Social Welfare Administration	Social Security Organization
11	35–50	Disability Benefits & Support	Social Security Organization
12	30–45	Social Care Management	Social Security Organization
13	40–55	Health Policy & Planning	Ministry of Health and Medical Education (MOHME)
14	35–50	Health Service Management	Ministry of Health and Medical Education (MOHME)
15	30–45	Community Health Programs	Iranian Red Crescent Society
16	45–55	Emergency & Rehabilitation Aid	Iranian Red Crescent Society
17	35–55	NGO Disability Advocacy	National Disability Support NGO
18	30–45	Rehabilitation Education	University Rehabilitation Faculty
19	40–50	Clinical Rehabilitation	Rehabilitation Specialty Hospital
20	35–50	Health Economics/Financing	University of Medical Sciences
21	45–60	Social Determinants of Health	Public Health Research Institute
22	30–45	Strategic Health Management	Ministry of Health and Medical Education (MOHME)

**Table 2:** Characteristics of FGD Participants (Two FGDs, total *n* = 24).

FGD group	Participant code	Age range	Field of activity	Organization
FGD 1	F1–F12	30–75	Rehabilitation providers, policymakers, caregivers	Mixed: State Welfare Organization, Rehabilitation Centers, NGOs
FGD 2	G1–G12	40–70	Health policy experts, managers, planners	Mixed: MOHME, Universities of Medical Sciences, Red Crescent Society

### 2.3 Data Collection

Data were gathered through semi‐structured, in‐depth interviews and document reviews. The primary researcher conducted the interviews and facilitated Focus Group Discussions (FGDs) between October 2023 and July 2024. Before each session, participants were briefed on the research objectives and assured of the confidentiality of their responses. Written consent was obtained for audio recording.

During the interviews, key topics were explored, and sessions were audio‐recorded with participants' permission. Detailed notes were also taken to supplement the recordings and ensure accuracy. To enhance data credibility, the researcher summarized key points during and at the end of each interview, allowing participants to confirm or clarify their statements.

All recorded interviews were transcribed verbatim for detailed analysis. Each session began with an open‐ended question addressing participants' perceptions of rehabilitation, associated challenges, and potential policy interventions.

### 2.4 Data Analysis

To analyze the data, a qualitative thematic analysis was conducted. Using an inductive approach, the text was initially reviewed, and relevant codes were assigned. Categories were then identified through systematic comparison. To ensure accuracy and consistency in coding, a peer review process was implemented. Data analysis was performed concurrently with data collection, allowing for continuous refinement. Once the coding process was completed and validated, key concepts were identified. MAXQDA version 22 was used for data analysis.

### 2.5 Trustworthiness of Data

To ensure the trustworthiness of the data, the study adhered to the four‐dimensional criteria proposed by Guba and Lincoln including credibility, dependability, confirmability, and transferability [22]. Credibility was established through several measures, including prolonged engagement in data collection, verification of interview data through peer and member checks, and cross‐validation of coding by two additional researchers who independently coded several initial interviews. Additionally, detailed notes were taken during the interviews, and the entire research process was thoroughly recorded.

Confirmability was ensured by systematically documenting the research process, maintaining a detailed record of the methodology, and examining contradictory or negative cases to understand inconsistencies in the findings. Expert opinions from individuals not directly involved in the research were also sought. Furthermore, the coding process was validated by two professors specializing in qualitative research, who reviewed the interview transcripts, assigned codes, and extracted categories to enhance reliability.

Transferability was considered by evaluating the extent to which the findings could be applied to other contexts. To support this, comprehensive documentation of the research methodology and findings was maintained, ensuring that other researchers could assess the applicability of the results in different settings.

## 3 Findings

The analysis of the findings indicates that considerations regarding rehabilitation in Iran are mainly related to four interconnected areas including the structure of the rehabilitation system, access to and availability of quality services, education and capacity building, and the role of key stakeholders. The policy interventions proposed by participants were grouped into four main categories including developing a policy framework, strengthening intersectoral and intrasectoral collaboration, expanding service and financial coverage, and enhancing human resource management. The themes and sub‐themes of rehabilitation considerations and related policy interventions in Iran heath system are presented in Tables 3 and 4.

**Table 3:** Themes and sub‐themes of rehabilitation considerations in Iran.

Rehabilitation system structure	Coordination across levels of care Education and research capacity in rehabilitation Integration of rehabilitation within the health system Representation of rehabilitation in primary healthcare Priority‐setting for rehabilitation services Governance and policy alignment
Availability and access to services	Cost considerations for users Distribution of human resources Quality and consistency of service delivery Service coordination across providers Therapeutic approaches and technologies Geographic distribution of rehabilitation professionals Social and cultural perceptions of rehabilitation Awareness of rehabilitation needs and benefits
Education and training	Challenges in Educational Fields Variability in Educational Program Quality Limited Academic Training Resources Need for Updating Educational Curricula and Plans
Actors and stakeholders	Role Definition Between Government and MoHME Sectoral Role Overlaps Stakeholder Engagement Levels Intersectoral Coordination Patterns Shared Framework for Collaborative Action Alignment of Public and Private Sector Roles

**Table 4:** Themes and sub‐themes of policy interventions for rehabilitation in Iran.

Developing policy framework	Defining and establishing a national policy and plan United governance and policy Identifying problems and priority setting
Intersectoral and intrasectoral cooperation	Stakeholders' engagement Promoting public‐private partnerships Defining role and effect of private sector, NGOs and community potential
Service& financial coverage	Insurance coverage Raising awareness and reducing stigma Cultural building
Human resources management	Equitable distribution of professionals Competency based education and training Need based curriculum development and revision Interdisciplinary teamwork in rehabilitation Quality of education and accreditation of rehabilitation disciplines

### 3.1 Rehabilitation System Structure

Rehabilitation services in Iran are influenced by the broader configuration of the national health system, which operates at multiple levels. The Ministry of Health and Medical Education (MoHME) oversees health services nationwide through a tiered structure that includes national, provincial, university, and primary healthcare (PHC) levels. At the primary level, Community Health Centers, Health Posts, and rural Health Houses serve as the first point of contact, while specialized and hospital‐based rehabilitation services are delivered at secondary and tertiary levels in teaching and provincial hospitals. The referral system, in principle, follows an ascending pathway from community and primary care to specialized rehabilitation units in hospitals or dedicated rehabilitation centers. However, pathways for referral and continuity of care vary across regions and service providers, and efforts to further standardize these linkages are ongoing.

Although a range of rehabilitation services exists across these levels, participants highlighted the need for a more clearly defined and centrally coordinated organizational structure within MoHME to support coherent planning and integration. Currently, the absence of a dedicated rehabilitation directorate means that responsibilities for policy, financing, and service management are distributed across different departments. This dispersion has led to parallel programs among key actors such as the MoHME, the State Welfare Organization, and the Iranian Red Crescent Society. Respondents noted that, while this multisectoral involvement reflects strong national commitment to rehabilitation, coordination mechanisms could be strengthened to ensure aligned priorities, shared standards, and optimal resource utilization.

One senior policymaker described this situation by stating:

“Rehabilitation is recognized across several organizations, and each plays an important role. However, developing a central coordinating body would help harmonize planning and reduce overlap, supporting more efficient use of resources.”

Participants also discussed the distribution of financing and strategic focus. While prevention and treatment have historically received significant investment, rehabilitation which forms the third level of care has opportunities for growth in terms of infrastructure development and workforce alignment. Strengthening rehabilitation within strategic health plans, and ensuring its integration into PHC and hospital‐based services, were identified as promising avenues.

A faculty member noted:

“When rehabilitation planning and financing can be coordinated more systematically across sectors, service delivery and continuity of care will be enhanced at all levels.”

Overall, participants emphasized that establishing a unified governance mechanism such as a national rehabilitation council or designated deputy office could support system‐wide planning, facilitate intersectoral collaboration, and reinforce rehabilitation's role within the continuum of care.

### 3.2 Availability and Access to Services

Participants noted that ensuring equitable availability and access to rehabilitation services remains an area in need of further attention within the national service delivery system. While a range of rehabilitation services is available across different levels of care, interviewees indicated that geographic distribution, financial accessibility, and referral pathways influence how different population groups ultimately experience service access.

Many respondents observed that rehabilitation facilities and specialized professionals are more frequently located in major urban centers, whereas some rural and peripheral areas have fewer service points or limited specialized expertise. They emphasized that further strengthening of referral linkages between primary care, secondary care, and specialized rehabilitation centers could help enhance continuity of care across regions.

A senior health manager explained:

“When referral pathways are clarified and consistently implemented, individuals in remote areas can be connected earlier and more efficiently to the services they require.”

Financial accessibility was also described as an important consideration. Participants highlighted that for individuals requiring long‐term or intensive rehabilitation, the extent of insurance coverage and reimbursement rates can influence continuity of care. Enhancing financial protection mechanisms particularly for people living with disabilities or chronic conditions was identified as an opportunity to improve equitable access.

A health insurance representative stated:

“Supporting families through more stable coverage can help ensure that those who need ongoing therapy can continue receiving it without facing disproportionate financial strain.”

In addition, respondents referred to the importance of provider payment structures and reimbursement tariffs in shaping service availability. They noted that adjustments in this area may support the expansion of high‐quality services, particularly in public facilities.

Another point raised by participants was the need for strengthening information systems for rehabilitation. The absence of a unified and comprehensive data system makes it challenging to estimate needs, plan resource distribution, and monitor utilization patterns. Developing integrated rehabilitation information systems was therefore identified as a strategic priority for improving planning and service delivery alignment.

As one MoHME official expressed:

“A shared national data platform would enable us to identify needs more accurately and align resources more effectively across regions.”

### 3.3 Actors and Stakeholders

Participants emphasized that strengthening collaboration and clarifying responsibilities among key actors is essential for improving coherence in Iran's rehabilitation system. They noted that multiple governmental and non‐governmental organizations including the MoHME, the Welfare Organization, and the Red Crescent Society are involved in rehabilitation service provision, yet their roles are not consistently defined within a shared coordination framework.

One policymaker described how these overlapping functions affect service navigation for beneficiaries:

“The Welfare Organization both supports and provides services, and its mandate intersects with that of the Ministry of Health. As a result, individuals may be uncertain about where to seek services, and resources are sometimes used in parallel rather than collaboratively.”

Participants noted that this fragmented structure can influence both access and quality, with some regions experiencing duplication of services while others, particularly rural or underserved areas, face shortages. They emphasized the importance of mechanisms that allow organizations to align planning and share responsibility in areas where coverage gaps are more prevalent.

Another theme concerned the limited engagement of the private sector in rehabilitation service delivery and equipment provision. Respondents explained that high service costs, partial insurance coverage, and financial uncertainties have made private sector participation more challenging in specialized fields such as audiology and advanced physiotherapy.

“Without clear financial incentives, many private providers are reluctant to invest in rehabilitation services. Public facilities alone cannot meet the increasing demand, which often results in delays or unmet needs.”

(Senior Manager)

Participants suggested that structured public–private partnerships and targeted subsidy models could encourage sustainable private sector involvement, helping expand access and diversify service capacity across regions.

### 3.4 Education and Training

Participants highlighted that deficiencies in rehabilitation education and workforce preparation have a direct impact on the quality and availability of services in Iran. A recurrent concern was that rehabilitation is not sufficiently integrated into core medical education.

*“*General practitioners rarely receive exposure to physical medicine or rehabilitation during their training. This lack of knowledge leads to fewer referrals and less appreciation for rehabilitation's importance.”

(Filed officer)

Interviewees also described significant barriers to workforce integration. Many rehabilitation graduates cannot secure positions in the public sector due to hiring freezes and budgetary constraints, while the privatization of hospitals has further limited students' clinical exposure, especially to diagnostic and therapeutic practices. Even in public institutions, investment in teaching infrastructure, specialized training centers, and faculty development remains inadequate, weakening the pipeline of skilled professionals.

Respondents further noted that financial barriers prevent many graduates from entering private practice. The high cost of essential equipment (e.g., for physiotherapy, audiology) and workspace rental makes establishing independent clinics unfeasible for most, which reduces the availability of community‐based rehabilitation services.

*“*Even those who graduate with strong skills often cannot afford the tools or clinic space to practice independently. They either leave the field or work in unrelated jobs.”

(Researcher)

Experts consistently underscored the urgent need for a national, structured framework to integrate rehabilitation services into Iran's health system. They emphasized that this framework must go beyond broad policy statements and provide clear governance structures, defined roles, and measurable priorities to address rising challenges, including the aging population, growing incidence of road traffic injuries, and the increasing prevalence of disabilities and chronic impairments.

Participants explained that the absence of such a framework currently results in fragmented planning and ad hoc programs, with rehabilitation often sidelined in national health strategies. A policymaker elaborated:

*“*Right now, rehabilitation is planned project by project. Without a national strategy and a coordinating authority, programs depend on temporary funding or individual champions rather than a sustained government commitment.*”*

Interviewees agreed that a robust framework should explicitly define human resource needs, standardized job descriptions, infrastructure requirements, and financial mechanisms at each level of service delivery. They also highlighted that dedicated funding streams and inter‐sectoral coordination mechanisms are critical for sustainable implementation.

Several participants proposed concrete institutional reforms. One suggested:

*“*To make policy‐making and program approval consistent, a steering committee for the national rehabilitation plan should operate under the High Council of Health and Food Security. In parallel, a dedicated national center for rehabilitation and social health should be created, reporting directly to the ministry.”

(Policy analyzer)

Experts further stressed that policy‐making must be grounded in research and education. Some advocated rebranding the existing Network Heath Management Center as a broader Health Management Center, expanding its mandate to include rehabilitation. Others recommended piloting regional programs with built‐in mechanisms for monitoring, evaluation, and continuous improvement, allowing the framework to remain adaptive and evidence‐driven over time.

### 3.5 Human Resources Management

Given the increasing prevalence of chronic illnesses, population aging, and the rising costs of long‐term rehabilitation care, participants highlighted human resources as an essential area requiring strategic strengthening within Iran's rehabilitation system. While demand for services continues to grow, the distribution and development of the rehabilitation workforce remain uneven, particularly in rural and underserved regions where access to services is already constrained.

Interviewees frequently emphasized the absence of a forward‐looking workforce planning framework that can anticipate future needs and align educational capacity and recruitment practices accordingly. Some noted that integrating rehabilitation concepts and referral pathways into both general medical and specialist training could support a broader understanding of rehabilitation across clinical fields.

“Planning for academic programs must match student intake with training infrastructure. Without this alignment, both the quality of education and workforce preparedness may be compromised.”

(Head of Educational Department)

Participants also underscored the value of structured fellowships and competency‐based continuing professional development to cultivate specialized expertise, particularly in areas such as neurorehabilitation, geriatric rehabilitation, and pediatric rehabilitation. However, several interviewees pointed out that administrative complexities and prolonged approval processes can delay the establishment of such programs.

“Specialized training programs are often proposed but remain in the approval pipeline for years, while patient needs continue to grow.”

(Rehabilitation Officer)

Some stakeholders suggested that facilitating more efficient approval processes, prioritizing educational program expansion in regions with adequate training infrastructure, and creating targeted incentives for professionals to practice in underserved areas could meaningfully strengthen the rehabilitation workforce.

### 3.6 Intersectoral and Intrasectoral Cooperation

Participants highlighted that the presence of multiple governmental and non‐governmental actors in the rehabilitation field creates complex institutional landscapes that require stronger coordination. They noted that while ministries such as the MoHME and the Ministry of Welfare have developed their own programs, these efforts often evolve in parallel with limited structured collaboration, leading to missed opportunities for shared planning and joint resource mobilization.

To address this, participants widely supported the establishment of a centralized interdepartmental coordination platform to harmonize policy development, financing strategies, and oversight of service delivery. Such a body, they suggested, would help clarify institutional roles and responsibilities, reduce overlaps, and support more integrated planning across sectors.

“There are several committees working on disability and rehabilitation, but they tend to function independently. A single authorized council with a defined mandate could align these activities and enhance the collective impact.”

(Mid‐level Program Coordinator)

Many interviewees recommended that this coordinating structure be anchored within the MoHME to ensure alignment with national health strategies and to facilitate more efficient use of health sector infrastructures and resources.

Beyond governmental collaborations, participants emphasized the need to formally recognize and systematize the contributions of private providers, NGOs, and community‐based organizations. They noted that these actors often extend access and introduce innovative approaches, yet their efforts are not consistently documented or integrated into national planning processes.

“The private sector and NGOs often help bridge service gaps, but without systematic monitoring and coordination, it becomes difficult to support their work or integrate their contributions into national strategies.”

(Case Manager)

Overall, participants stressed that strengthening intersectoral and intrasectoral collaboration presents an important opportunity to enhance coherence, reduce fragmentation, and expand equitable access to rehabilitation services.

### 3.7 Service and Financial Coverage

Participants emphasized that strengthening financial protection and service coverage is essential for promoting equitable access to rehabilitation services, particularly for older adults and people with disabilities in low‐income groups. They noted that addressing these needs requires an integrated approach that combines preventive care, financial support mechanisms, and social assistance.

Several interviewees highlighted the value of early identification and proactive planning, suggesting that screening for health risk factors and developing regional action plans for high‐risk elderly populations could help facilitate timely interventions and lessen the need for prolonged and costly rehabilitation in later stages. In addition, respondents underscored the importance of updating and regularly disseminating a national health indicators atlas specific to older adults to enable informed, age‐sensitive resource allocation.

“Without an up‐to‐date picture of health risks among the elderly, planning remains reactive rather than preventative. An updated atlas would help target resources more effectively.”

(Senior Health Manager)

Expanding comprehensive care packages and insurance coverage for vulnerable groups, especially persons with disabilities, was repeatedly described as a policy priority. Participants referred to the existing disability rights legislation that mandates insurance coverage for both physical and mental rehabilitation services. However, they noted that the implementation of this policy has been uneven, and many individuals continue to face out‐of‐pocket expenses.

“The law guarantees rehabilitation coverage, but in practice, many families still pay directly for services, which can create financial strain.”

(Insurance Officer)

Another commonly noted recommendation was strengthening caregiver and family training. Many respondents suggested that allocating a substantial portion of service package resources to caregiver education could improve rehabilitation outcomes, enhance continuity of care at home, and help reduce long‐term system costs.

Finally, participants stressed the importance of ensuring access to mental health support for vulnerable groups including individuals with disabilities, their caregivers, and at‐risk older adults through coordinated action between the Ministry of Health and partner organizations.

## 4 Discussion

This study aimed to examine the key considerations and policy interventions related to positioning rehabilitation services as a priority within Iran's health system. In Iran, the current level of integration in rehabilitation services is influenced by the broader structural features of the health system. These structural conditions have contributed to some variation in the coordination and continuity of service delivery among the organizations involved in providing care. Additionally, aspects related to the governance of rehabilitation services, as well as financing and insurance coverage, play an important role in shaping the overall accessibility of these services. Internationally, countries such as Sweden and Norway have successfully integrated rehabilitation services within their PHC systems, benefiting from well‐structured and highly coordinated health infrastructures [23].

Monitoring the provision and delivery of rehabilitation services remains an important area of consideration within Iran's health system. In the current context, organizations such as the Welfare Organization, the Red Crescent, and the Ministry of Health and Medical Education each contribute to the delivery of rehabilitation services; however, these efforts take place across different institutional frameworks. Consequently, coordination and unified oversight in this sector have been limited.

In many countries, the Ministry of Health typically assumes stewardship for rehabilitation services, while in Iran, a substantial portion of this responsibility is assigned to the Welfare Organization. Understanding this organizational structure is essential for informing future planning aimed at enhancing coherence and alignment across rehabilitation service delivery.

The World Health Organization referred to this in its 2019 report, emphasizing the importance of the health sector's oversight of the rehabilitation sector [24]. The results of our study indicate that although the primary and secondary levels of care in the Iranian health system have developed significantly, the third level of service delivery, namely rehabilitation, has been neglected, which has led to gaps in the service delivery system [25].

Insufficient insurance coverage for rehabilitation services was also noted in this study as an important area of consideration, which may contribute to increased out‐of‐pocket payments, particularly among households with greater financial vulnerability who are more likely to require such services. Considering the diversity of rehabilitation needs across different population groups, it is important that these services be appropriately prioritized and delivered in a cost‐effective manner, alongside efforts to enhance insurance support [11]. The result of the study conducted in Iran showed that households requiring rehabilitation services were 6.7 times more likely to experience catastrophic expenditures compared to those without such services [26]. This challenge has been consistently highlighted in both global [27, 28, 29] and local literature [30]. There is a need and a necessity to examine the share of rehabilitation in the country's insurance package and to improve rehabilitation services by allocating a budget and increasing it annually, as well as to improve the quality of these services. If this measure is not institutionalized in the health system soon, the health system will face numerous problems in the near future. It is also important to note that the provision of home rehabilitation services can reduce the additional costs of hospitalization [31].

Currently, rehabilitation services for people with disabilities in Iran are delivered through several executive agencies, each operating within its own framework, which has resulted in varying degrees of alignment in policy and implementation. To support the more effective provision of rehabilitation services, strengthened and coordinated governance may be beneficial. This could include promoting balanced legislation, joint policy‐making, and enhanced collaboration within and between key partner institutions such as the Ministry of Health and Medical Education (MoHME), the Ministry of Cooperatives, Labor and Social Welfare, the National Welfare Organization, the Red Crescent Society, the Organization for Exceptional Education, and the Martyrs and Veterans Affairs Foundation.

To help ensure that individuals in need have consistent access to rehabilitation services, the integration of these services across all levels of the health system, particularly within primary health care, is an important consideration. Such integration can support the availability of services within communities and help maintain continuity of care [32]. On the other hand, NGOs in our country cooperate in the field of rehabilitation under the name of charities in specific areas such as job creation and preserving the cultural identity of persons with disabilites. Also, charitable organizations for the disabled can access the latest achievements in adaptation through communication with non‐governmental organizations at the international level and transfer these matters to the relevant institutions in the country [33].

Human resources stewardship can be achieved by training and making multidisciplinary rehabilitation human resources available, improving rehabilitation training courses, creating a comprehensive information system to facilitate the follow‐up of people in need of rehabilitation services, improving referral pathways, establishing centers for identifying the disabled and incapacitated, and creating and accessing a database of the disabled, separated by disability and incapacity. A precise definition and separation of rehabilitation services are among other suggested interventions. Furthermore, under the Population Increase Law of 2021 [34], evaluating the policy's impact on infants with abnormalities and disabilities, and their subsequent rehabilitation needs, is imperative. Increasing public awareness of the importance of preventing disabilities caused by congenital conditions is equally vital.

Overall, it is necessary to make it a priority to improve the health of all members of society, not just people with disabilities. This will help achieve universal health coverage in Iran.

## 5 Conclusion

Iran's rehabilitation system presents areas that may benefit from evidence‐based strategies to review and strengthen existing structures, with the aim of ensuring an effective response to the needs of persons with disabilities and individuals requiring rehabilitation services. Prioritizing these considerations can support policymakers and planners in progressing toward the goals of universal health coverage in Iran.

Given the diversity and specialized nature of rehabilitation services, as well as the importance of broadening service coverage and enhancing access for target groups, fostering interdisciplinary collaboration among the various relevant organizations and institutions involved in rehabilitation is essential.

## Author Contributions

Leila Doshmangir conceptualized the study, extracted and interpreted the data, and drafted and revised the manuscript. Bahram Amirshakeri contributed to the study design, data extraction and interpretation, and assisted in revising and preparing the final version of the manuscript. Maryam Ghiassipour contributed to the study's conception and interpretation and provided critical feedback on the manuscript. Roghayeh Khabiri contributed to drafting, revising, and preparing the final version of the manuscript, as well as editing the article. Reza Majdzadeh contributed to the study conceptualization, data interpretation, and manuscript revision. All authors have read and approved the submitted and final revised version of the manuscript.

## Ethics Statement

This study was approved by Medical Ethics Committee at Tabriz University of Medical Sciences (Ethics approval No: IR.TBZMED.REC.1403.898). All authors have read and approved the final version of the manuscript, corresponding authors had full access to all of the data in this study. All authors takes responsibility for the integrity of the data and the accuracy of the data analysis.

## Conflicts of Interest

The authors declare no conflicts of interest.

## Transparency Statement

The lead author Bahram Amirshakeri affirms that this manuscript is an honest, accurate, and transparent account of the study being reported; that no important aspects of the study have been omitted; and that any discrepancies from the study as planned (and, if relevant, registered) have been explained.

## Data Availability

All data generated or analyzed during this study are included in this article and its supplementary information files are available from the corresponding author on reasonable request.
